# Right-first bilateral transcervical single-port robotic esophagectomy: a cadaveric feasibility study toward a safer extrapleural strategy

**DOI:** 10.1007/s00464-026-12792-8

**Published:** 2026-04-20

**Authors:** Olga Meier, Olga Greenberg, Justin Kemper, Yulia Brecht, Franziska Renger, Hiroyuki Daiko, Peter P. Grimminger

**Affiliations:** 1 Department of General, Visceral and Transplant Surgery, University Medical Center of the Johannes Gutenberg-University Mainz, Langenbeckstr. 1, 55131 Mainz, Germany; 2 Hirslanden Clinic Zurich, Zurich, Switzerland; 3 Intuitive Surgical Inc., Sunnyvale, CA USA; 4 Department of Esophageal Surgery, National Cancer Center Hospital, Tokyo, Japan

**Keywords:** Esophagectomy, Transcervical, Single-port robot, Extrapleural, Recurrent laryngeal nerve, Mediastinal lymphadenectomy

## Abstract

**Background:**

Left transcervical extrapleural esophageal mobilization can avoid pleural entry and one-lung ventilation but is limited by challenging exposure of the right paratracheal compartment and a relevant risk of left recurrent laryngeal nerve (RLN) injury. Prior bilateral transcervical approaches have been constrained by instrument collisions and limited caudal reach.

**Methods:**

In a fresh-frozen cadaver, a right-first, bilateral transcervical single-port (SP) robotic strategy was performed with sequential right-then-left cervical docking under low-pressure pneumomediastinum. Feasibility endpoints were predefined: (i) reproducible access to the paratracheal and subcarinal planes, (ii) identification and preservation of the RLNs, (iii) maintenance of an extrapleural corridor to the diaphragmatic hiatus, and (iv) collision-free workflow.

**Results:**

Right-sided transcervical docking enabled near-circumferential thoracic esophageal mobilization with early identification of the right RLN and systematic mediastinal lymphadenectomy, including reliable exposure of the subcarinal station. The SP configuration allowed stable traction with two working instruments, facilitating controlled dissection without relevant internal or external collisions. The subsequent left-sided phase focused on completion of residual mobilization and targeted lymphadenectomy along the left RLN with minimal traction and limited energy application. All predefined feasibility criteria were met.

**Conclusions:**

A threshold-guided (~ 85%) right-first bilateral transcervical SP robotic approach is feasible in a cadaveric model. This strategy improves access to the right paratracheal nodal stations and may reduce traction on the left RLN by shifting traction-intensive steps to the right. Prospective clinical translation is warranted to benchmark RLN outcomes, nodal yield, pleural integrity, and recovery against current transcervical and transthoracic techniques.

**Graphical Abstract:**

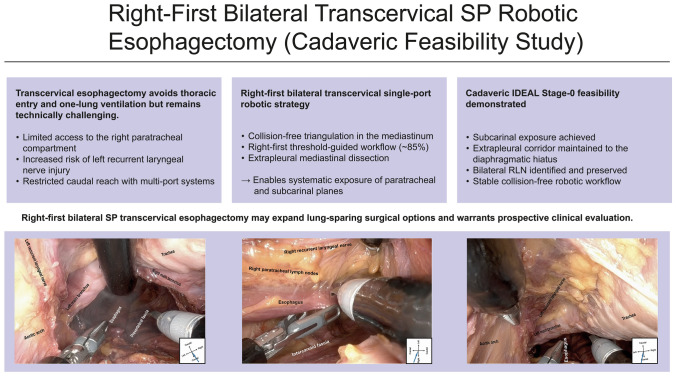

**Supplementary Information:**

The online version contains supplementary material available at 10.1007/s00464-026-12792-8.

Transthoracic esophagectomy—whether open, thoracoscopic, or robotic—requires pleural entry and single-lung ventilation to access the mediastinum [1, 2]. Although these approaches provide excellent exposure, they are associated with relevant pulmonary morbidity, particularly in patients with compromised respiratory reserve or hostile pleural conditions. In such settings, one-lung ventilation and pleural adhesiolysis increase the risk of postoperative pneumonia, respiratory failure, and prolonged recovery [1–4].

Transcervical extrapleural approaches represent an alternative pathway to thoracic esophageal mobilization and mediastinal lymphadenectomy while avoiding pleural entry and preserving lung mechanics [5–8]. Early mediastinoscopy-assisted techniques demonstrated the feasibility of this route and showed favorable respiratory outcomes compared with transthoracic approaches [3]. However, these techniques were constrained by limited visualization, rigid instrumentation, and restricted maneuverability within the narrow mediastinal corridor, particularly in the paratracheal and subcarinal regions.

As a result, traditional transcervical techniques were associated with an increased risk of recurrent laryngeal nerve (RLN) injury—most notably on the left side, where the nerve follows a longer intrathoracic course and loops beneath the aortic arch before ascending within the tracheoesophageal groove [3, 4]. Traction during ventral esophageal mobilization and lymphadenectomy has been identified as a key mechanism underlying left-sided RLN palsy in transcervical approaches.

The introduction of robotic systems substantially expanded the technical potential of transcervical esophagectomy. Wristed instruments, stable three-dimensional optics, and improved ergonomics enabled more precise dissection around critical mediastinal structures and facilitated systematic lymphadenectomy [1, 2, 5, 7, 8]. Subsequent bilateral transcervical robotic series, particularly from high-volume Asian centers, demonstrated acceptable nodal yields and favorable pulmonary outcomes, confirming that bilateral cervical access improves exposure of the right paratracheal compartment while allowing deliberate handling of the left RLN [1, 2, 4]. Nevertheless, conventional multi-port robotic systems remain limited by external arm collisions and restricted cranio-caudal reach, often preventing reliable access beyond the carina from a cervical approach alone.

The da Vinci Single-Port (SP) system was developed to address these limitations. By delivering three fully wristed instruments and a flexible three-dimensional endoscope through a single compact trocar, the SP platform reduces external crowding and restores true intracorporeal triangulation within confined spaces such as the mediastinum [5, 8]. Cadaveric and early clinical studies have demonstrated improved ergonomics, smoother instrument articulation, and enhanced access to subcarinal planes compared with multi-port systems [5, 8].

Parallel advances in surgical anatomy research have further refined transcervical robotic esophagectomy. Detailed anatomical mapping has defined reproducible extrapleural dissection planes along the intercarotid and prevertebral fasciae posteriorly and the visceral fascia along the tracheoesophageal groove anteriorly, while identifying key hazard structures including the thoracic duct, azygos vein, aortic arch, pulmonary vessels, and the RLNs [6, 9]. These frameworks emphasize delayed, deliberate RLN dissection and minimizing traction within the high-risk left tracheoesophageal groove.

Building on these anatomical and technological developments, we evaluated a right-first, threshold-guided bilateral transcervical SP robotic strategy, advancing to the left side only after approximately 85% of circumferential esophageal mobilization and planned mediastinal lymphadenectomy had been completed from the right. This sequence is based on two principles: first, concentrating traction-intensive maneuvers on the right side—where the RLN is shorter and courses more ventrally—may reduce mechanical stress on the left RLN; second, a right-sided entry provides superior access to the right paratracheal nodal stations and a favorable trajectory for proximal esophageal mobilization.

This study follows the IDEAL framework for surgical innovation and represents a Preclinical (Stage 0) cadaveric feasibility investigation. Its objective was to assess the technical feasibility, anatomic reproducibility, and ergonomic stability of a right-first bilateral transcervical SP robotic approach as a foundation for subsequent clinical translation.

## Objectives

To evaluate the feasibility of a right-first, threshold-guided (~ 85%) bilateral transcervical SP robotic approach with predefined endpoints:

- Reproducible access to paratracheal and subcarinal dissection planes;
- Bilateral identification and preservation of RLNs;
- Maintenance of an extrapleural corridor toward the diaphragmatic hiatus;
- Stable, collision-free ergonomics under pneumomediastinum.

## Methods

### Study design and setting

This procedure-development study was performed on a fresh-frozen male cadaver at IRCAD (Institut de Recherche contre les Cancers de l’Appareil Digestif), a certified European surgical training and research center equipped with advanced multiple robotic platforms and high-fidelity simulation infrastructure.

To maximize clinical transferability, the laboratory workflow mirrored SP-RACE practice, including patient positioning, docking geometry, instrument configuration, and standardized Lexicon insufflation parameters [5]. A prospective lab log documented each step in real time—positioning/docking sequences, any internal or external collisions, insufflation settings, instrument changes, exposure endpoints (paratracheal, subcarinal, hiatal, and extrapleural continuity), and troubleshooting measures—to ensure reproducibility and to capture ergonomics relevant to subsequent clinical translation.

### Participants and expertise

The study was conducted by a multidisciplinary team with substantial experience in esophageal surgery. The two lead operators—one European and one Asian esophageal surgeon—had extensive experience with multi-port and single-port robotic transthoracic esophagectomy, as well as mediastinoscopic and single-port/multi-port robotic transcervical approaches, including the development of novel esophagectomy techniques [2, 3, 5, 7]. A European fellow in robotic esophageal surgery primarily assisted at the table and performed approximately 50% of the bilateral lymphadenectomy along the recurrent laryngeal nerves (the second phase of each RLN dissection) under the direct supervision of one of the expert surgeons. The fellow had limited prior SP console experience (training exposure only), substantial bedside experience in SP esophagectomy (both subcostal and transcervical approaches), and two years of console experience in multi-port robotic esophagectomy. Two da Vinci system specialists provided advanced technical support for robotic setup, docking optimization, and instrument calibration. An IRCAD staff member with extensive cadaver-lab experience assisted with logistics, specimen preparation, and laboratory coordination.

### Technique setup: patient positioning, cervical incisions, and insufflation protocol

The cadaver was positioned supine with a small shoulder roll to achieve gentle cervical extension. The head was slightly rotated to the left during the right-sided phase and to the right during the left-sided phase, with the table kept level throughout the procedure.

**Fig. 1:**
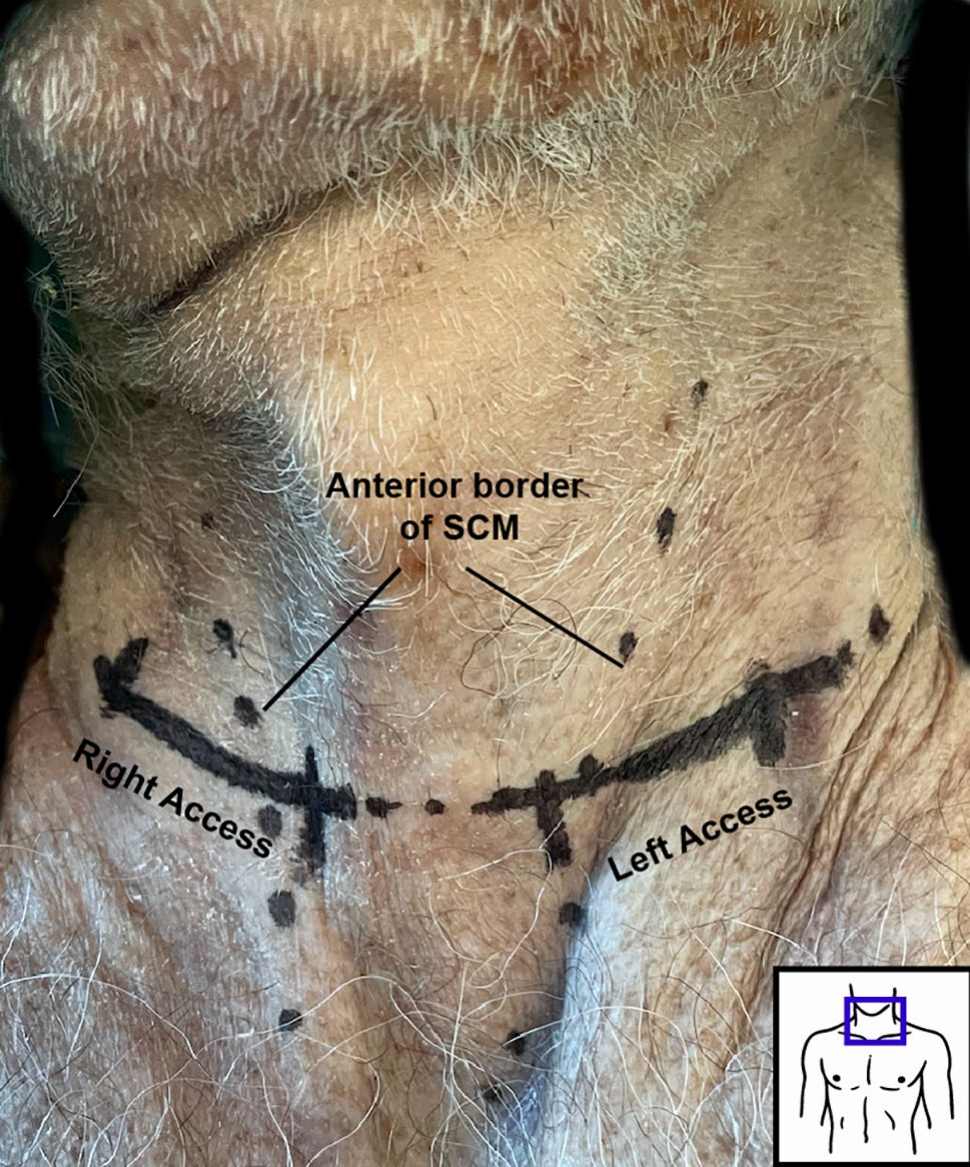
Cervical access sites. Cervical access sites. The initial right cervical skin-crease incision (~ 3 cm) is made in a natural fold approximately two fingerbreadths above the clavicle. Upon completion of the right-sided dissection, the balloon trocar is intentionally left in place to preserve mediastinal seal and exposure during the contralateral phase. A symmetric left-sided cervicotomy is then created, and a second balloon trocar is inserted. *SCM* Musculus sternocleidomastoideus

Cervical access was established at the beginning of each transcervical phase through bilateral ~ 3 cm skin-crease incisions positioned two fingerbreadths above the clavicle to optimize exposure and cosmesis (Fig. 1). Subcutaneous tissue and platysma were incised in line with the skin incision, and short subplatysmal flaps were elevated cranially and caudally to widen the field. The sternocleidomastoid (SCM) was exposed; the superficial layer of the deep cervical fascia was opened along the anterior border of the SCM, which was then retracted laterally with preservation of the external jugular vein. The infrahyoid muscles were identified and gently retracted medially. The lower pole of the thyroid was mobilized and retracted medially; the inferior thyroid veins were identified and ligated. To enlarge the corridor, the omohyoid was divided.

A wound-retractor type single-port access device (da Vinci SP Access Port Kit, Intuitive Surgical, Sunnyvale, CA, USA) was then inserted. The internal (lower) ring was anchored deep to the strap muscles medially and beneath the SCM laterally; the external ring was intentionally left partially untightened and positioned ~ 2 cm above the skin to improve maneuverability and visualization at the cervical level. The operative window was defined laterally by the carotid sheath (common carotid artery, internal jugular vein, vagus nerve) and medially by the visceral compartment. The chamber of the access port was then attached to the wound retractor’s external clamping ring.

Pneumomediastinum was established using a 12 mm Lexicon Insuflow trocar inserted into the chamber of the Access Port Kit at 8 mmHg CO₂, with brief increases to 15 mmHg for deeper mediastinal windows. A high-flow regimen stabilized the field, ensured continuous smoke evacuation, and minimized tissue distortion while maintaining image clarity.

**Fig. 2:**
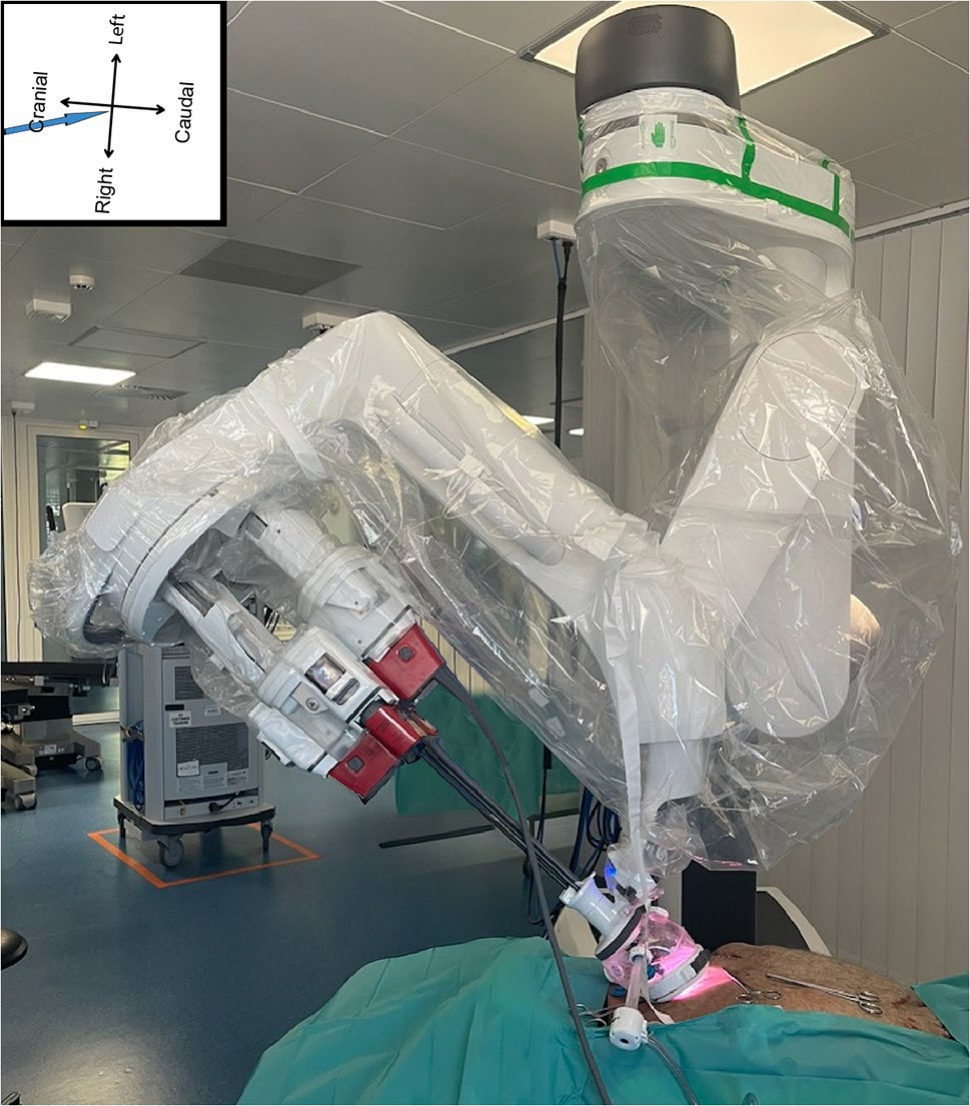
SP system setup. A balloon-type cervical access port is placed with the inner ring seated beneath the sternocleidomastoid laterally and the strap muscles medially. The da Vinci Single-Port system is docked from the head end, with the boom positioned above the head, thereby optimizing the cranio-caudal working angle and preserving space for a concurrent abdominal team. Although not implemented in the laboratory setting, this arrangement is necessary for a two-team approach in clinical practice. Instrument layout: fenestrated bipolar forceps (Arm 1), flexible 3D endoscope, Cadiere forceps (Arm 3), and monopolar curved scissors (Arm 4), with the endoscope in a “cobra” top-down orientation

The single-port instrument setup comprised fenestrated bipolar forceps (Arm 1, surgeon’s left), a Cadiere forceps (Arm 2, central–inferior), and monopolar curved scissors (Arm 3, surgeon’s right). The 3D endoscope, mounted centrally in a top-down orientation, was typically positioned in a “cobra” configuration to optimize visualization of the upper mediastinum and subcarinal planes (Fig. 2). No instrument changes were required throughout the procedure.

### Right-first, threshold-guided strategy

In this study, the transcervical phase was initiated on the right side using a “right-first, threshold-guided” strategy, enabling systematic dissection of cervical structures and the upper and lower mediastinum down to the esophageal hiatus under direct three-dimensional robotic visualization.

Feasibility endpoints specified the target anatomical structures to be identified and exposed, thereby guiding the surgical steps to be performed via the appropriate transcervical approach.

### Right-sided phase (Figs. 3, 4, 5, 6)

**Fig. 3:**
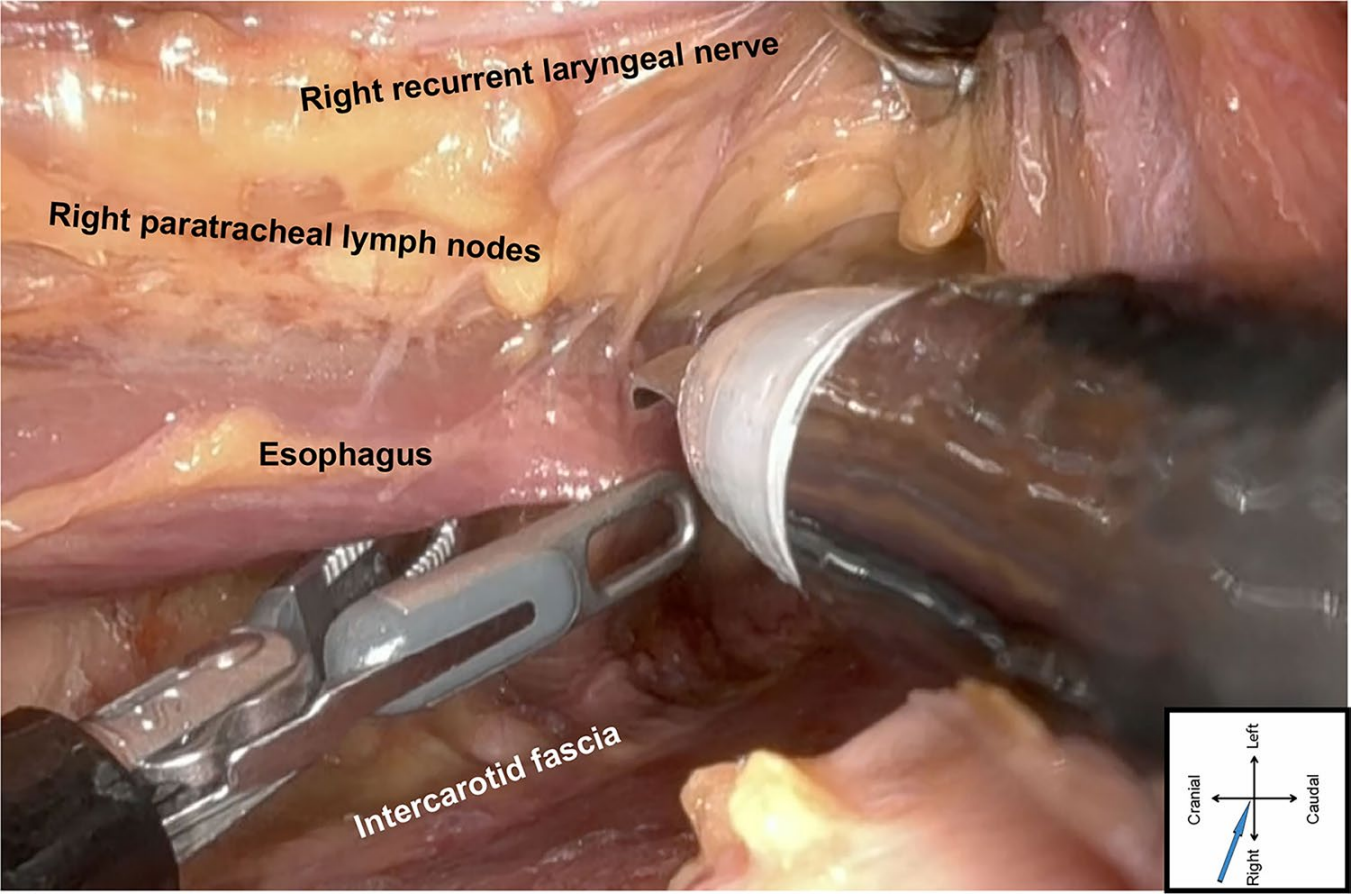
Right transcervical dissection and safe entry into the ventral plane. The right-sided posterior dissection is already advanced: the intercarotid fascia has been developed to establish the posterior plane. Superiorly, the right RLN is exposed with paratracheal lymph nodes draping the trachea, while the esophagus remains covered only by visceral fascia. The visceral fascia is incised at the tracheoesophageal groove at a safe distance from the RLN, beginning at the level of the superior thoracic aperture to initiate the ventral dissection. *RLN* recurrent laryngeal nerve

**Fig. 4:**
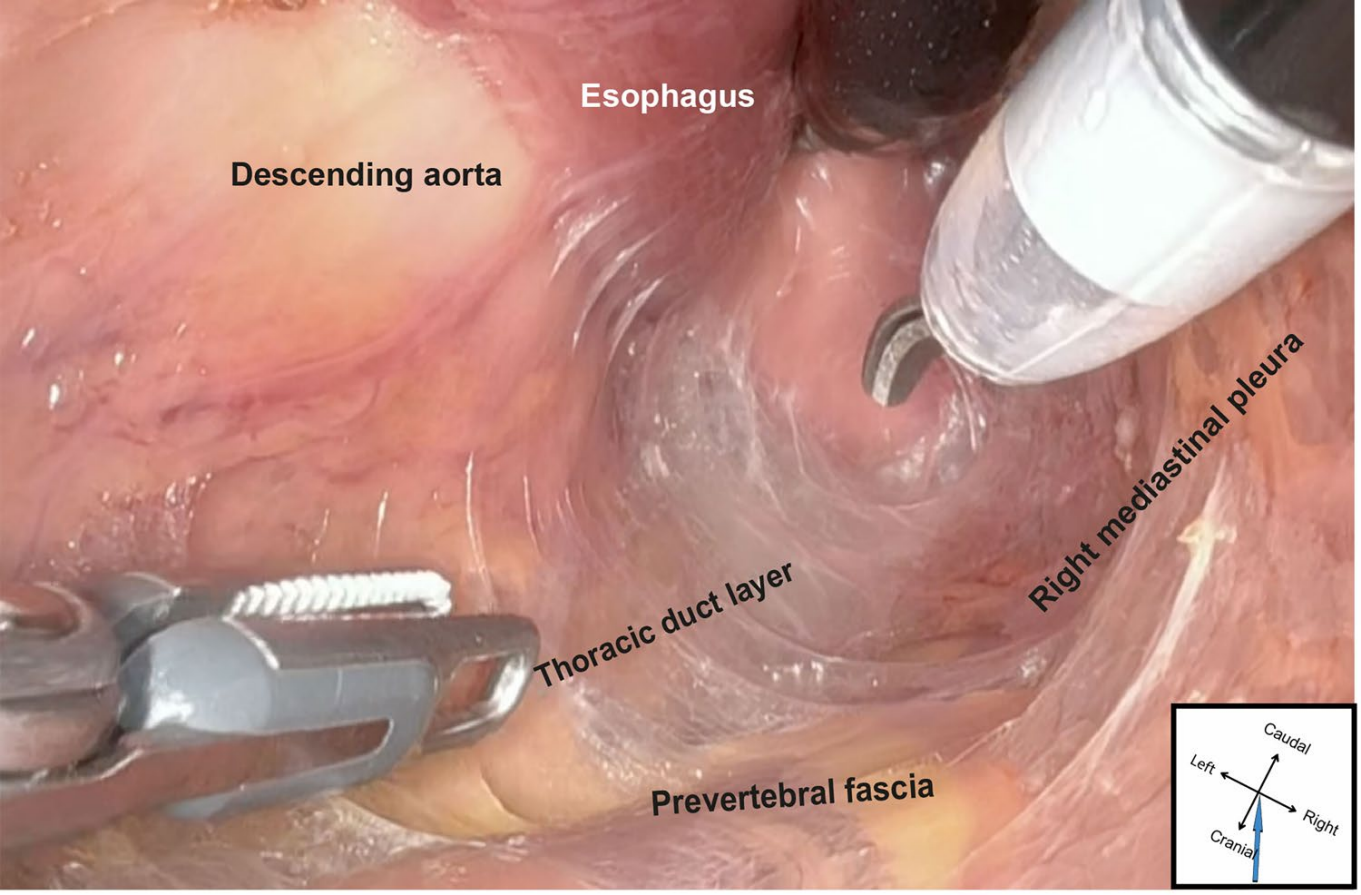
Progression of the posterior dissection. The posterior dissection proceeds along the prevertebral fascia, while the esophagus remains attached anteriorly to the tracheobronchial tree and the posterior pericardium. The descending aorta is identified on the left. On the inferior left, the thoracic duct plane is left intact to protect the duct. On the right, the mediastinal pleura is followed as the lateral dissection plane

**Fig. 5:**
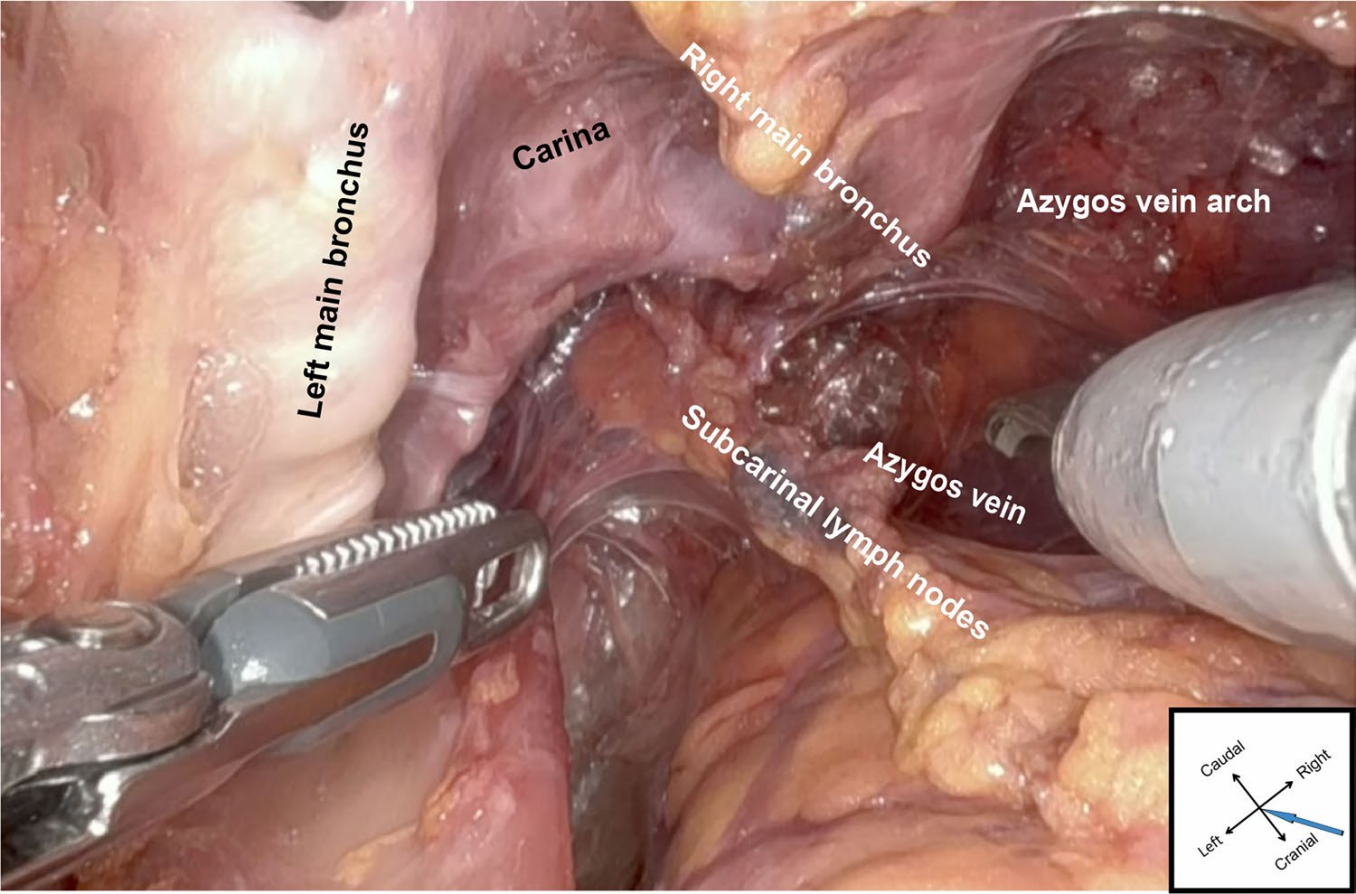
Continuation of the anterior dissection and subcarinal exposure. The anterior dissection proceeds along the pars membranacea of the trachea and main bronchi, exposing the carina and the supracardiac region. A subcarinal and retrocardiac lymphadenectomy is performed en bloc with the esophagus, leaving the lymphatic tissue attached to the esophageal wall. To the right of the esophagus, the azygos arch is seen coursing from posterior to anterior, while the azygos vein runs parallel along its right-lateral border

**Fig. 6:**
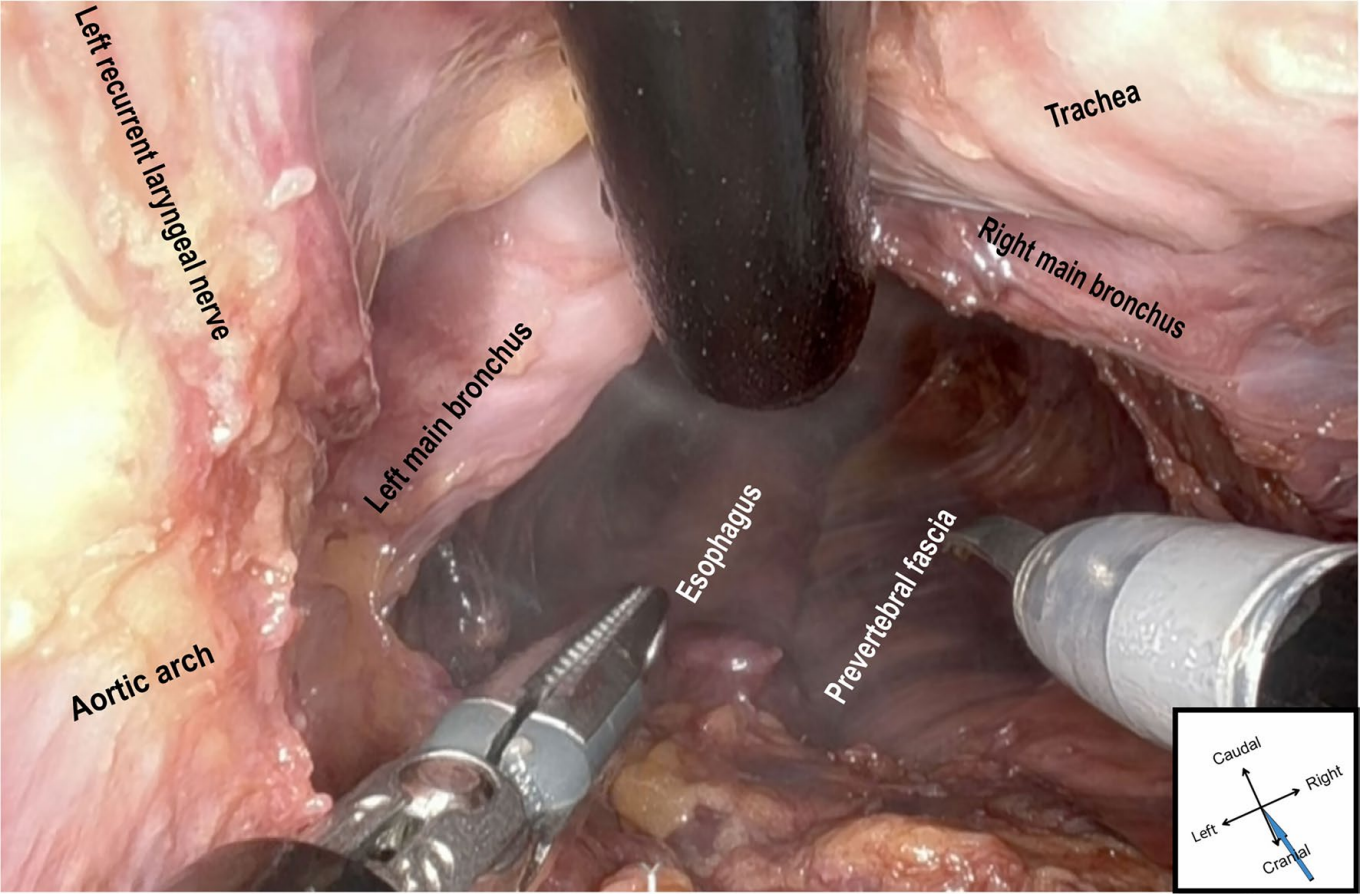
Completion of the right transcervical esophageal mobilization and key landmarks. From the right approach, the carina and both main bronchi are exposed anteriorly; the trachea lies superiorly on the right, and the aortic arch is seen on the left. The proximal segment of the left RLN is identified between the superior border of the left main bronchus and the inferior margin of the aortic arch. Posteriorly, the plane is developed along the prevertebral fascia. Caudal to the carina, the esophagus is mobilized circumferentially (360°) with completion of the lymphadenectomy in the lower mediastinum. Cranial to the carina, mobilization is limited to approximately 85% of the circumference, intentionally preserving the fibrous attachments between the cartilaginous trachea and the esophageal adventitia on the left—as well as the visceral fascia within the left tracheoesophageal groove. *RLN* recurrent laryngeal nerve

1. *Right carotid sheath (lateral boundary)*: To delineate the lateral limit of dissection by identifying the common carotid artery, internal jugular vein, and vagus nerve.
2. *Right recurrent laryngeal nerve (RLN)*: To identify and continuously visualize it along its course slightly ventral to the right tracheoesophageal groove; the proximal segment is to remain attached to the trachea with its small tracheal branches to prevent traction injury until the final step of the right-sided phase.
3. *Visceral fascia (enveloping the trachea and esophagus)*: Incise the fascia along the right tracheoesophageal groove at a safe distance from the right RLN—located adjacent to the visceral fascia and slightly toward the trachea—and expose the layer between the anterior esophageal wall and the posterior tracheal wall. This establishes the primary entry corridor for the anterior esophageal dissection plane down to the carina.
4. *Trachea*: To divide the fibrous connections between the right esophageal border and the tracheal cartilage sharply, and to bluntly separate the pars membranacea from the esophagus; the left margin of the tracheoesophageal plane is not to be dissected in this phase to protect the left RLN. This plane serves as the ventral dissection layer.
5. *Intercarotid fascia (posterior boundary of the cervical compartment)*: The fascia located between the two carotid sheaths defines the initial posterior dissection plane. When preserved, this layer protects the thoracic duct and the cervical sympathetic trunk, which run between the intercarotid fascia and the prevertebral fascia.
6. *Right parietal pleura*: Identify the right mediastinal pleura laterally to demarcate the lateral boundary of the upper mediastinum just caudal to the superior thoracic aperture.
7. *Prevertebral fascia (posterior mediastinal limit)*: To define—and then follow—the posterior limit of dissection where the intercarotid fascia tapers caudally.
8. *Thoracic duct*: Identify the duct between the azygos vein and the aorta, posterolateral to the esophagus on the left, and preserve it within its thin fascial layer.
9. *Azygos vein*: Expose and preserve the azygos arch on the right—approximately at the level of the aortic arch—separating it from the esophagus, to serve as a superior landmark of the middle mediastinum.
10. *Right bronchial artery*: Identify the artery—medial to the azygos arch and right-lateral to the esophagus, arising from the intercostobronchial trunk—and preserve it whenever feasible.
11. *Right vagus nerve*: To identify the vagus along the right tracheoesophageal groove, beginning in the upper mediastinum and then coursing posterior to the right main bronchus; its cardiac and pulmonary branches are to be visualized and possibly preserved.
12. *Aortic arch and descending thoracic aorta*: Visualize the aortic arch to the left of the esophagus, superior to the left main bronchus, and approximately at the level of the azygos arch; then, more caudally, identify the descending thoracic aorta coursing posteriorly and left-lateral to the esophagus, forming the left-posterior dissection plane down to the hiatus.
13. *Left recurrent laryngeal nerve (proximal segment)*: Identify the nerve at the aortic arch as it loops beneath the ligamentum arteriosum and ascends toward the left tracheoesophageal groove; right-sided visualization of this left RLN segment facilitates its subsequent identification and dissection on the left.
14. *Aortopulmonary window*: Expose the window between the inferior border of the aortic arch and the superior border of the left main bronchus, with the left pulmonary artery in depth; identify and, when indicated, dissect the aortopulmonary lymph nodes.
15. *Carina and main bronchi*: Expose the posterior wall of the distal trachea, both main bronchi, and the carina, with identification and dissection of the subcarinal lymph node station.
16. *Posterior pericardium*: To identify the pericardium over the left atrium as the anterior boundary of the deepest mediastinal plane, with dissection of retropericardial lymph nodes.
17. *Superior and inferior pulmonary veins (right and left)*: Use the veins as deep caudal landmarks, delineating the distal ventrolateral dissection plane.
18. *Esophageal hiatus*: To define the distal endpoint of the right transcervical mediastinal preparation.

### Left-sided phase (Figs. 7, 8)

**Fig. 7:**
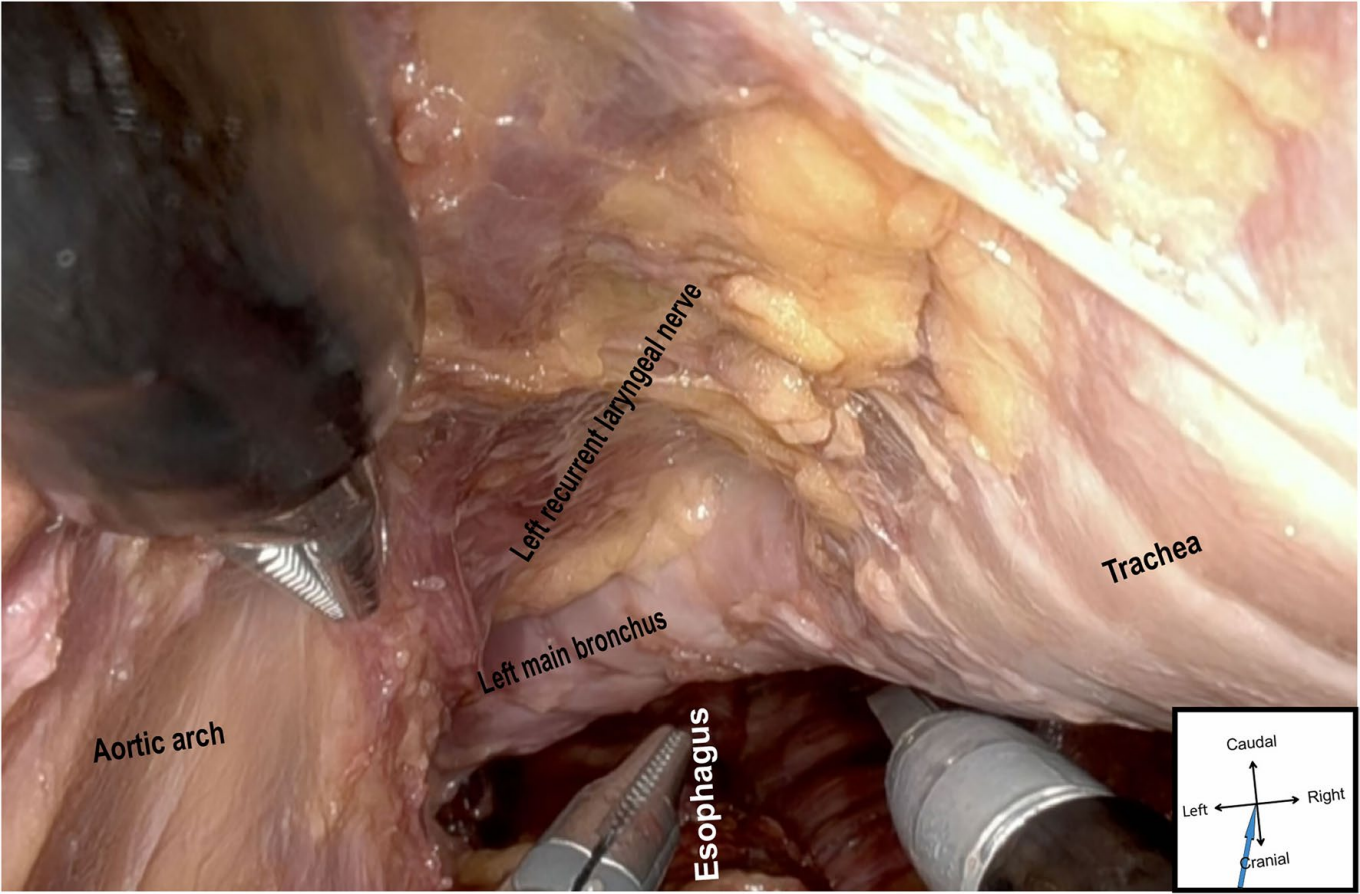
Early left-sided phase: nerve-sparing dissection. At the start of the left-sided phase, the proximal segment of the left RLN—already exposed during the prior right-sided dissection—is identified. The left RLN is kept under continuous visualization while the left esophageal border is dissected off the left side of the trachea at a safe distance from the nerve. During this phase, the RLN remains protected by surrounding soft tissue. The aortic arch is visible on the left. In the superior field, the left main bronchus and supracarinal trachea are seen; inferiorly, the mobilized esophagus is visible. *RLN* recurrent laryngeal nerve

**Fig. 8:**
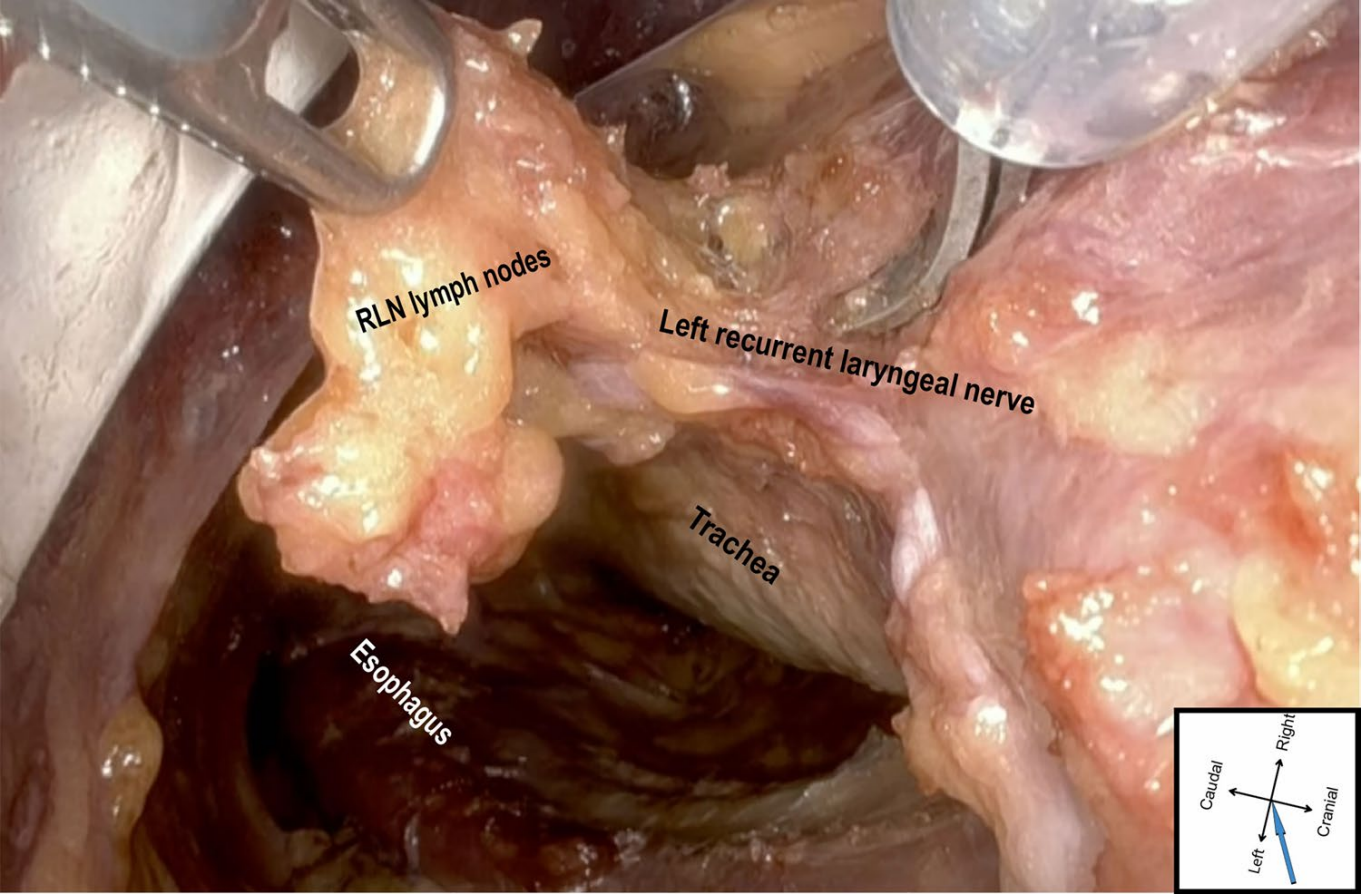
As the final step of the transcervical dissection, a left paratracheal lymphadenectomy along the recurrent laryngeal nerve is performed with minimal thermal energy, used only for targeted coagulation of exposed perineural vessels

1. *Left carotid sheath (lateral boundary)*: To delineate the lateral limit of dissection by identifying the common carotid artery, internal jugular vein, and vagus nerve.
2. *Left RLN*: Perform left paratracheal lymphadenectomy at the end of the left transcervical phase to minimize overall left RLN trauma.

### Data sources

Procedural specifics and timestamps were derived from contemporaneous lab notes, raw video from the da Vinci system, and external images from the pre- and post-robotic phases. A comprehensive procedural video accompanying this manuscript illustrates docking geometry, right-first mediastinal progression, subcarinal exposure, hiatal reach, and bilateral RLN dissection.

## Results

All predefined feasibility endpoints were met. On the right side, incision was performed at 08:56 and docking was completed at 09:10 (incision-to-docking: 14 min). On the left side, incision was performed at 10:20 and docking was completed at 10:30 (incision-to-docking: 10 min).

The right transcervical phase was completed in 55 min, including 15 min dedicated to lymphadenectomy along the right RLN. All target anatomical structures were successfully identified, and the extrapleural corridor was maintained down to the esophageal hiatus.

The left transcervical phase required 25 min, including 14 min for targeted lymphadenectomy along the left RLN. The nerve was continuously visualized, preserved anatomically, and handled with minimal traction.

Only one minor ergonomic adjustment (head repositioning) was required to avoid external contact between the access port and the chin. No internal or external robotic collisions occurred, and both RLNs remained anatomically intact.

### Surgeon feedback

Survey responses indicated a high level of procedural satisfaction. RLNs’ identification was reported as straightforward, and lymphadenectomy could be performed safely and effectively, owing to the ergonomic exposure and instrument maneuverability.

## Discussion

This cadaveric study demonstrates the technical feasibility and ergonomic stability of a right-first, bilateral transcervical single-port robotic approach for extrapleural thoracic esophageal mobilization with systematic mediastinal lymphadenectomy. The strategy addresses two central limitations of transcervical esophagectomy: restricted access to the right paratracheal compartment and the risk of traction-related injury to the left recurrent laryngeal nerve.

The findings align with and extend prior work showing that robotic transcervical approaches can overcome the camera instability and straight-instrument limitations of mediastinoscopy while preserving the advantages of the extrapleural route—namely, avoiding thoracic entry and one-lung ventilation [1, 2, 4, 5, 7].

The left RLN is particularly susceptible to traction during ventral esophageal mobilization via a left cervical approach. Accordingly, historical transcervical series have reported higher rates of left-sided RLN palsy, which are often mitigated by continuous intraoperative nerve monitoring [1–4]. Concentrating traction-intensive dissection steps on the right, where the RLN is shorter and courses more ventrally, is anatomically rational and may reduce mechanical stress on the left RLN [1, 3].

Our stepwise, right-first approach follows modern anatomic models that define avascular planes—intercarotid to prevertebral posteriorly and the visceral fascia ventrally along the tracheoesophageal groove—and identify key hazard structures, including the thoracic duct, azygos vein, aortic arch, and bronchial artery [6, 9]. These references frame the mediastinum in three “boxes” (thoracic inlet, upper mediastinum, subcarinal), supporting controlled cranio-caudal progression and safe navigation of critical zones [6, 9].

Compared with multi-port robotic systems, the SP platform introduces three fully wristed instruments and a flexible 3D endoscope through a single compact cervical port. This configuration minimizes both internal and external arm collisions. It restores true intracorporeal triangulation within the narrow mediastinal corridor—particularly advantageous for precise work around the paratracheal and deep subcarinal regions [5, 7, 8]. Recent SP “how-to” guides and the first SP-RACE case series have reported efficient mediastinal mobilization with adequate nodal yields, less instrument interference, and smoother articulation of the 6-mm SP instruments [5]. These ergonomic and exposure advantages were consistently reproduced in our controlled cadaveric setting.

Earlier multi-port transcervical robotic studies confirmed reliable access to the paratracheal and carinal planes but often reported caudal limitations inferior to the carina due to collision constraints inherent to multi-port systems [1, 2, 4]. In our model, the right-first SP sequence maintained a stable extrapleural corridor down to the diaphragmatic hiatus with only a single instance of minor head repositioning and right-sided redocking to optimize orientation. This reach supports the concept that the SP system’s coaxial flexibility can bridge the anatomic gap between the carinal and hiatal zones.

Consistent with prior SP cadaveric and clinical series, minor adjustments in head position and port height (“off-skin” ring position) effectively prevented head–hub proximity and external collisions. Stable insufflation with the Lexicon system at 8 mmHg CO₂, with brief boosts to 15 mmHg, maintained clear visualization and minimized tissue distortion throughout the procedure.

Existing bilateral mediastinoscopic and multi-port robotic cohorts from high-volume Asian centers have demonstrated acceptable nodal yields, low pulmonary morbidity, and safe bilateral RLN handling [1–4]. However, these systems remain limited by arm collisions and restricted cranio-caudal reach. The SP configuration directly addresses both issues. Furthermore, recent bilateral transcervical robotic studies using multi-arm systems emphasized that reproducibility depends on deliberate nerve strategy and anatomic discipline—principles that are integral to our right-first, threshold-guided concept [1, 2, 4].

From a practical standpoint, since the abdominal phase of esophagectomy is currently performed laparoscopically in most centers, a transcervical route that allows subcarinal lymphadenectomy from above is highly valuable. While a simultaneous abdominal robotic phase is technically feasible, it requires two robotic systems, as demonstrated in prior reports. This nodal station represents a standard oncologic target in Europe, yet it remains challenging to access transabdominally via laparoscopy.

In clinical application, specimen extraction may be performed transcervically when a posterior mediastinal conduit route is planned, or transabdominally if a retrosternal conduit is preferred, depending on the institutional reconstruction strategy.

Within the IDEAL framework, this work represents a Pre-IDEAL (Stage 0) preclinical investigation defining procedural steps, anatomic safety planes, and ergonomic feasibility prior to clinical translation [10, 11]. The logical next phase will be a Stage 1–2a development study with standardized metrics, including nodal yield per station, pleural integrity, bilateral RLN function, postoperative pneumonia, and length of stay, documented under a transparent protocol.

For patients unfit for transthoracic esophagectomy or with hostile pleura, a right-first bilateral SP transcervical approach may offer an extrapleural, lung-sparing alternative while maintaining oncologic completeness. It enhances access to the right paratracheal and subcarinal lymph nodes and allows controlled, low-traction left-sided completion while preserving the RLN. Early SP experiences and detailed anatomy-based frameworks support the transition from preclinical to clinical testing. Future comparative studies should benchmark this strategy against existing multi-port and single-port techniques, as well as conventional minimally invasive transcervical and transthoracic approaches, with a focus on RLN outcomes, nodal yield, and respiratory morbidity.

### Open problems and gray zones

Despite encouraging preclinical feasibility, several precautions must be considered before clinical implementation of a right-first bilateral transcervical SP robotic esophagectomy.

### Hemorrhage control and conversion strategy

Although the extrapleural route avoids pulmonary parenchymal injury and one-lung ventilation, major mediastinal bleeding remains a potential concern. Relevant hazard structures include the azygos vein, bronchial arteries, aortic branches to the esophagus, and vessels within the aortopulmonary window.

While robotic magnification and stable traction may facilitate precise dissection, control of sudden major bleeding through a purely transcervical approach may be technically challenging. Because the patient is positioned supine and thoracic access may be limited—particularly in the presence of hostile pleura—initial hemostatic management may require transcervical or transabdominal control strategies.

Therefore, early clinical translation should incorporate predefined emergency conversion protocols, including readiness for thoracic access. Prospective evaluation of intraoperative bleeding risk and conversion thresholds will be essential during IDEAL Stage 1–2a development. Importantly, the anticipated bleeding risk does not appear higher than in established transcervical mediastinoscopic or single-port left-sided approaches.

### Energy modality and instrument limitations

In this cadaveric model, monopolar curved scissors were used for most of the dissection due to current SP platform instrument availability. A two-bipolar dissection strategy represents a feasible alternative and may enhance hemostatic safety, particularly during early clinical implementation.

Although bipolar graspers provided adjunctive hemostasis, advanced SP-compatible vessel-sealing devices are not yet commercially available. Future iterations of SP instrumentation—including dedicated sealing devices and expanded energy options—may further enhance procedural safety.

### Anatomic variability and patient selection

Technical feasibility appears to depend more strongly on cervical anatomy than on body mass index alone. Factors such as short neck length, limited cervical extension, or increased pretracheal soft tissue may reduce working space and instrument angulation.

While no strict BMI cutoff is proposed, early clinical application should emphasize careful anatomical assessment and gradual case selection within experienced robotic esophageal programs.

### Oncologic completeness and nodal yield

Although subcarinal and paratracheal stations were well exposed in this cadaveric model, oncologic adequacy—defined by nodal yield per station and dissection quality—must be validated clinically. Direct comparison with established transthoracic robotic approaches will be necessary to confirm non-inferiority in mediastinal lymphadenectomy.

### Maximum caudal reach

In the present model, the extrapleural corridor was maintained to the diaphragmatic hiatus without collision-related limitations. However, thoracic length variability across patients could not be systematically quantified in this single cadaveric case. Future cadaveric and clinical studies should evaluate measurable caudal reach and define anatomical limits of the SP platform.

### RLN functional outcomes

Anatomical preservation of both recurrent laryngeal nerves was achieved in this model. However, functional outcomes—particularly transient versus permanent RLN palsy rates—can only be assessed in clinical trials with standardized laryngoscopic follow-up. The proposed right-first threshold-guided strategy aims to reduce traction exposure to the left RLN, but this hypothesis requires prospective validation.

### Tracheobronchial traction and force control

In addition to recurrent laryngeal nerve protection, traction on the trachea and main bronchi represents a relevant consideration during transcervical mediastinal dissection. In the absence of haptic feedback in the robotic system, traction must be carefully controlled based on visual cues alone. In our approach, retraction is applied in a controlled and intermittent manner, guided by tissue deformation and anatomical planes. Notably, the SP platform generates lower mechanical force compared to multi-port systems, which may reduce the risk of excessive traction. Based on our prior clinical experience with left-sided SP transcervical approaches, no tracheal or bronchial traction-related injuries have been observed. Nevertheless, this aspect requires further evaluation in clinical studies.

### Learning curve and dissemination

Although reproducibility was supported by fellows performing approximately 50% of bilateral RLN lymphadenectomies under supervision, structured training pathways will be required. Safe dissemination should include:

o High-volume robotic esophageal programs
o Standardized procedural algorithms
o Bilateral RLN monitoring
o Formal proctoring during early cases

Broader adoption should await multi-institutional experience.

### Clinical translation

The next step should be prospective clinical development (IDEAL Stage 1–2a) with standardized metrics including bilateral RLN function (with objective laryngoscopy), nodal yield per station, pleural integrity, postoperative pulmonary complications, and recovery endpoints compared with established transcervical and transthoracic approaches.

## Conclusions

In a cadaveric model, a threshold-guided (~ 85%) right-first bilateral transcervical SP robotic esophagectomy strategy is feasible and ergonomically stable. The configuration improves exposure of right paratracheal nodal stations and may mitigate left RLN traction exposure by relocating traction-intensive steps to the right. Prospective clinical studies are warranted to benchmark RLN outcomes, nodal yield, pleural integrity, and postoperative recovery.

## Supplementary Information

Below is the link to the electronic supplementary material.

Supplementary file1 (MP4 476479 KB)

## Data Availability

Data supporting the findings are available from the corresponding author upon reasonable request.
